# CVNet: Lightweight Cross-View Vehicle ReID with Multi-Scale Localization

**DOI:** 10.3390/s25092809

**Published:** 2025-04-29

**Authors:** Wenji Yin, Baixuan Han, Yueping Peng, Hexiang Hao, Zecong Ye, Yu Shen, Yanjun Cai, Wenchao Kang

**Affiliations:** School of Information Engineering, PAP Engineering University, Xi’an 710086, China; 20210058@ntit.edu.cn (W.Y.); hbx911wj@163.com (B.H.); yzc6666@yeah.net (Z.Y.); dreamofcaiyj@163.com (Y.C.);

**Keywords:** re-identification, cross-view, lightweight network

## Abstract

Cross-view vehicle re-identification (ReID) between aerial and ground perspectives is challenging due to limited computational resources on edge devices and significant scale variations. We propose CVNet, a lightweight network with two key modules: the multi-scale localization (MSL) module and the deep–shallow filtrate collaboration (DFC) module. The MSL module employs multi-scale depthwise separable convolutions and a localization attention mechanism to extract multi-scale features and localize salient regions, addressing viewpoint variations. DFC employs a dual-branch design comprising deep and shallow branches, integrating a filtration module optimized via neural architecture search, a collaboration module, and lightweight convolutions. This design effectively captures both unique and shared cross-view features, ensuring efficient and robust feature representation. We also release a new CVPair v1.0 dataset, the first benchmark for cross-view ReID, containing 14,969 images of 894 vehicle identities, offering results of traditional and lightweight methods. CVNet achieves state-of-the-art performance on CVPair v1.0, VehicleID, and VeRi776, advancing cross-view vehicle ReID. The dataset will be released publicly.

## 1. Introduction

The advancement of aerial photography has transformed road monitoring, creating new opportunities for vehicle ReID [1,2,3,4]. However, cross-view vehicle ReID between aerial and ground perspectives remains challenging due to differences in viewing angles, significant scale variations, and the limited computational resources of edge devices.

Vehicle ReID methods typically rely on backbone networks with classification heads. While convolutional neural networks (CNNs) have advanced feature extraction through global and local aggregation strategies, they face limitations. Global pooling layers dilute discriminative local features, and spatial division or part-based detection methods demand extensive annotations and high computational costs. To address these issues, studies have integrated CNNs [5] with graph neural networks (GNNs) [6] for improved feature relationship modeling. Transformers [7] enhance global and local feature learning but are constrained by high computational demands and limited adaptability to multi-scale, low-resolution inputs. Lightweight backbones [8] offer efficiency but struggle to maintain robust performance in cross-view ReID tasks. These challenges underscore the need for architectures that balance efficiency, multi-resolution input handling, and cross-view alignment.

**Figure 1:**
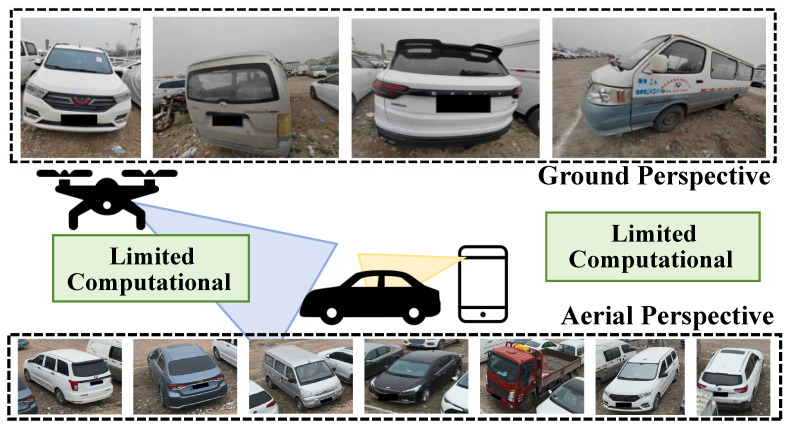
Constrained computational resources on unmanned aerial platforms and resolution variations caused by perspective changes.

As illustrated in Figure 1, several critical challenges hinder the advancement of cross-view vehicle ReID systems. First, aerial capture devices, such as Jetson Nano and other edge computing platforms, often suffer from limited computational resources, making it infeasible to deploy traditional heavy ReID models. This constraint underscores the need for lightweight architectures capable of balancing efficiency and accuracy, similar to recent advancements in efficient conditional generation frameworks [9,10]. Second, the inherent differences in image properties caused by heterogeneous capture devices—including drones, mobile phones, and ground surveillance cameras—introduce significant variations in scale, resolution, and viewpoint. These inconsistencies are further exacerbated by perspective changes, leading to vehicle images with diverse sizes, shapes, and aspect ratios. Existing multi-scale feature fusion methods like Feature Pyramid Networks (FPNs) [11] partially address this issue, but often struggle to preserve critical structural details in cross-resolution scenarios. To this end, robust feature extraction methods that can adaptively capture cross-view contextual information are essential, drawing inspiration from multi-level conditioning strategies in generative tasks [12,13]. Third, the lack of large-scale datasets specifically designed for cross-view vehicle ReID remains a significant bottleneck. Although person ReID has seen rapid progress driven by the availability of diverse cross-view datasets [14,15], vehicle ReID research is limited by limited resources. This gap hampers the development of models capable of handling perspective shifts, resolution discrepancies, and domain variations. Addressing this challenge may benefit from bipartite-aware similarity learning [16] and contrastive learning strategies [17], which have been proven effective in other cross-domain retrieval tasks.

To overcome these issues, we propose CVNet, a lightweight architecture specifically designed for cross-view vehicle ReID. CVNet incorporates two innovative modules: the multi-scale localization (MSL) module and the deep–shallow filtrate collaboration (DFC) module. The MSL module employs multi-scale depthwise separable convolutions alongside localization attention mechanisms to enable precise regional localization through multi-scale feature extraction and fusion. Meanwhile, the DFC module adopts a dual-branch design, leveraging lightweight convolutions discovered via neural architecture search (NAS) to extract both shared and unique features across perspectives. CVNet incorporates two novel modules: the MSL module and the DFC module. The MSL module leverages multi-scale depthwise separable convolutions and localization attention mechanisms to precisely extract regional features through multi-scale feature extraction and information fusion. The DFC module adopts a dual-branch structure with lightweight convolutions discovered through NAS to process cross-view images. This design enhances scene understanding and strengthens the robustness of deep and shallow feature representations. Additionally, we introduce CVPair v1.0, the first benchmark dataset tailored for cross-view vehicle ReID. CVPair v1.0 contains 14,969 images of 894 vehicle identities, offering results of traditional and lightweight methods. The main contributions of this work are as follows:

- We propose CVNet, a lightweight network with only 4.4M parameters, achieving state-of-the-art performance.
- We devise the MSL module, which enhances precise regional positioning through multi-scale feature extraction and fusion, tailored for complex scenarios.
- We develop the DFC module, designed to extract both shared and unique features across diverse perspectives, improving cross-view feature representation.
- We present CVPair v1.0, the first benchmark dataset for cross-view vehicle ReID, offering results of traditional and lightweight methods.

## 2. Related Work

### 2.1. Datasets for Vehicle ReID

**Ground–Ground Vehicle ReID Datasets:** As shown in Table 1, the VERI-Wild [18] and VehicleID [19] datasets are two prominent public repositories designed for the task of vehicle ReID. These datasets serve as valuable resources for researchers aiming to develop and evaluate vehicle recognition algorithms. The VehicleID [19] dataset comprises 221,763 images across 26,267 unique vehicle identities, whereas the VERI-Wild [18] dataset is substantially larger, encompassing 416,314 images of 40,671 distinct vehicle identities. The VehicleID [19] dataset is characterized by its simplicity and uniformity, with the majority of images featuring frontal or rear views of vehicles. In contrast, the VERI-Wild [18] dataset presents a more sophisticated challenge due to its inclusion of diverse perspectives and environmental conditions, such as varying lighting and instances of partial occlusion. This complexity introduces a layer of difficulty for researchers, particularly when addressing issues related to occlusion and viewpoint changes. The VERI-Wild [18] dataset’s enhanced authenticity and complexity render it particularly well suited for studies focused on real-world vehicle ReID scenarios. Its comprehensive nature provides a more robust platform for the development of algorithms that must contend with the variability and unpredictability inherent in field environments.

**Aerial–Aerial Vehicle ReID Datasets:** These are designed to align with scenarios where vehicle images are captured by multiple unmanned aerial vehicles (UAVs). Notable datasets within this domain include VRAI [20], VeRi-UAV [21], VRU [22], and UAV-VeID [23], each contributing unique attributes to the field of vehicle ReID. The VRAI [20] dataset stands out for its comprehensive annotations that extend beyond mere perspectives. It provides detailed part annotations for each vehicle instance, facilitating the distinction of specific vehicle features. This includes classifications for color (class 9), vehicle type (class 7), and the presence of a sunroof, bumper, spare tire, and luggage rack. The image resolution captured by the drones may vary due to fluctuations in altitude, leading to a broader range of resolutions compared to the VehicleID [19] dataset. The VeRi-UAV [21] dataset comprises a total of 81 videos, from which 2158 scene images with a resolution of 4096 × 2160 were meticulously screened. The dataset encapsulates 17,515 images of 454 vehicles, capturing a diverse array of weather and lighting conditions, and preserving natural settings. Each vehicle image is not only meticulously tagged with its ID and viewpoint but also enriched with spatio-temporal information, including video ID and scene sequence data. The UAV-VeID [23] dataset is distinguished by its realism, with drones operating at altitudes ranging from 15 to 60 m, yielding a spectrum of vehicle image proportions. The camera’s vertical angle varies from 40 to 80 degrees, introducing a variety of viewing angles. The images are collected under unconstrained, natural conditions, without artificial modification, thus defining a more authentic vehicle ReID task. Lastly, the VRU [22] dataset is an expansive compilation within this category, featuring footage from four flying drones. With an altitude range of 15 to 60 m, the dataset contains 172,137 images of 15,085 vehicles. It is structured into one training set and three distinct test sets—small, medium, and large—to accommodate varying scales of evaluation.

**Aerial–Ground Vehicle ReID Datasets:** The AG-ReID [14] and G2APS [15] datasets represent the current state of Aerial–Ground person ReID datasets. The AG-ReID [14] dataset comprises 21,893 images across 388 unique identities, offering a rich tapestry of data captured by two cameras situated in a bustling outdoor environment. Utilizing DJI drones, the dataset benefits from a dynamic range of altitudes between 15 and 45 m, providing a diverse array of perspectives and backgrounds through both aerial and fixed-camera footage. The G2APS [15] dataset, focused on personal identification, presents a substantial collection of 2644 individual identities and an extensive compilation of 260,559 bounding boxes. This dataset’s encompasses flight altitudes from 20 to 60 m, ground surveillance cameras positioned approximately 2.0 m above the ground, and a diverse set of perspectives, attitudes, and patterns captured by drones as they record individuals in various poses. The datasets simulate a wide spectrum of real-world conditions, characterized by significant view changes between query and gallery images, and enriched by complex environmental scenes.

However, to the best of our knowledge, there exists a notable absence of datasets dedicated to vehicle ReID across aerial and ground camera perspectives. Our work aims to take the inaugural step towards addressing this gap, paving the way for future research and technological advancements in the field.

### 2.2. Neural Architecture Search Task

NAS is increasingly favored by the computer vision community for its characteristics of automated architecture design and is usually composed of three main parts: search space, search strategy, and performance evaluation. The role of NAS is to automate the search for the optimal network architecture, which not only saves researchers a lot of time and effort but also helps to discover innovative network structures that human experts may overlook. With NAS, the network can be customized for specific tasks, improving the performance and efficiency of the model. Compared to traditional methods of designing network architectures by hand, NAS is able to quickly explore a large number of network possibilities and find an architecture that is better suited to a specific task. In the ReID task, NAS in particular shows its advantage. The ReID task requires the model to recognize and match pedestrians under different cameras, which requires the model to be able to capture robust and discriminating features. NAS can customize the network for ReID tasks and automatically search for network structures that are more suitable for capturing multiple scales, perspectives, and features, thus improving the accuracy and robustness of recognition.

At present, Progressive Neural Architecture Search (PNAS) [24] proposes a progressive search strategy to search architectures by gradually increasing the complexity of the network. Efficient Neural Architecture Search (ENAS) [25] optimizes network architecture through reinforcement learning, which significantly improves search efficiency. Differentiable Architecture Search (DARTS) [26] treats network architecture search as a differentiable process, allowing optimization using gradient descent methods. Search network architecture using neural architecture search with reinforcement learning (NASNet) [27] especially improves the efficiency of the network.

However, most of the current NAS strategies do not consider the special task of air–ground cross-view ReID, and the challenges such as view changes and resolution differences in the task are not fully considered in the NAS search process, so it is necessary to design or adjust the search strategy specifically.

## 3. CVPair v1.0 Dataset

**Figure 2:**
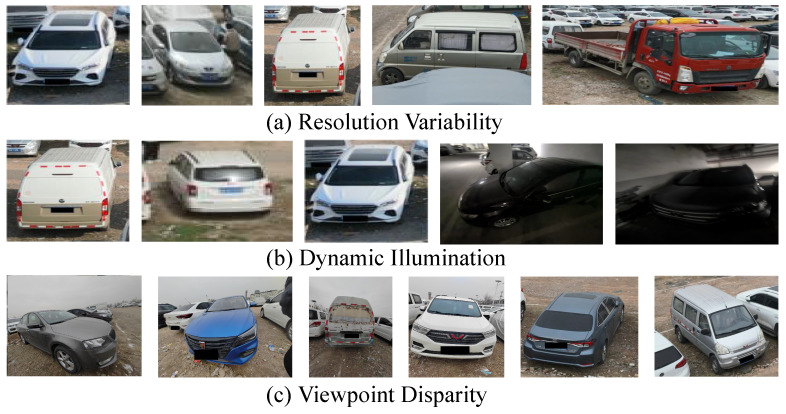
Our dataset CVPair v1.0 faces the challenges of resolution variability, dynamic illumination and viewpoint disparity.

To address the lack of datasets designed for cross-view vehicle ReID tasks, as shown in Figure 2, we release the CVPair v1.0 dataset, the first benchmark tailored for cross-view vehicle ReID. The dataset construction involved four key processes—collection, filtration, annotation, and split—ensuring both quality and relevance. CVPair v1.0 encompasses a diverse range of challenging scenarios, including open-air and underground environments, and provides a comprehensive evaluation benchmark for this underexplored task.

**Collection.** The CVPair v1.0 dataset, comprising 14,969 images of 894 vehicles, introduces a unique challenge in vehicle ReID by spanning both open-air and underground environments. It includes aerial and ground-level views, capturing diverse perspectives that significantly increase the complexity of air-to-ground ReID.

**Filtration.** During dataset construction, we implemented stringent filters to guarantee data quality and relevance. Our trained review teams removed irrelevant content like background noise and objects, as well as images with technical flaws like overexposure and blurriness.

**Annotations.** To boost vehicle detection accuracy in high-altitude aerial photos, we manually curated a dataset. Annotators extracted vehicle images from drone footage. We used a cross-annotation process with two annotators verifying each other’s work for accuracy. The dataset, CVPair v1.0, was divided into categories ‘A’ for aerial images and ‘G’ for ground images.

**Split.** The dataset is divided evenly, with 50% allocated for training and 50% for testing. The training set comprises 391 identities and 7448 images, while the test set includes 1006 query images and 6515 gallery images. Model performance is evaluated under two scenarios: aerial-to-ground (A2G) and ground-to-aerial (G2A).

**Challenge.** From Figure 2, the key attributes of our dataset are as follows: first, resolution variability, resulting from the different capture devices used for aerial and ground images; second, dynamic illumination, such as features observed in the underground garage scenario, where fluctuating lighting conditions blur vehicle images, creating real-world recognition challenges; finally, viewpoint disparity, with top-down images offering features distinct from ground-level perspectives.

## 4. Methodology

### 4.1. Overall

**Figure 3:**
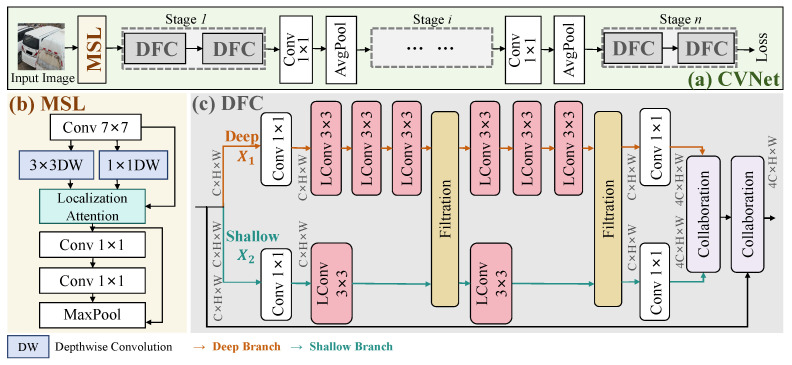
Overview of the CVNet architecture. The multi-scale localization (MSL) module extracts multi-scale features and employs attention mechanisms for salient region localization. The deep–shallow filtrate collaboration (DFC) module adopts dual branches (deep and shallow) to capture shared and unique cross-view features. LConv consists of convolution, batch normalization, and ReLU activation.

From Figure 3, we propose CVNet, a lightweight network with only 4.4M parameters, featuring the MSL and DFC modules. The network starts with a 0.09 M MSL module, which employs multi-scale depthwise separable convolutions and a localization attention mechanism to extract multi-scale features and localize salient regions. Image features are then processed through *n* stages, with average pooling between each to reduce complexity. Each stage contains two DFC modules, which refine features using a dual-branch mechanism to handle multi-perspective images. By combining the MSL’s extraction with the DFC’s filtrate collaboration, the network captures expressive features, improving accuracy for images from varying perspectives. This design boosts both parameter efficiency and enhances the model’s robustness to variations in perspectives.

### 4.2. Multi-Scale Localization

Traditional feature extraction methods struggle with images captured at varying perspectives, leading to performance issues. Increasing network depth to compensate adds a significant computational burden, especially on edge devices of limited computational resources. As shown in Figure 3, the proposed MSL module addresses this challenge by combining depthwise convolution (DW) with localization attention modules in a lightweight design. This module can be seamlessly integrated into any backbone for enhanced vehicle ReID. Initially, features are extracted via a convolution operation and split into three branches. The first branch applies a 1×1 DW for cross-channel fusion, while the second employs a 3×3 DW for deeper feature extraction. These multi-scale features, along with the third branch, are fed into the localization attention module, which adjusts weights based on input to overcome the limitations of standard convolutions.

The localization attention module encompasses a global average pooling operation, followed by a sigmoid activation function, and culminates in the multiplication of the resultant weights with the original feature maps. This architectural choice enhances the network’s representational capacity, enabling it to focus on the most significant features. After localization attention, two convolutional layers with max pooling reduce the feature map size while increasing displacement invariance. A residual structure ensures efficient gradient flow and prevents degradation. MSL’s combination of convolutions, DW, localization attention, and pooling structures offers an efficient, lightweight feature extractor. Positioned before the backbone, it enriches feature representation with minimal additional parameters, significantly boosting extraction capabilities.

### 4.3. Deep–Shallow Filtrate Collaboration

Our proposed DFC module, illustrated in Figure 3, incorporates convolution, filtration, and collaboration modules. The input feature is represented as $X\in R^{C\times H\times W}$, where *C*, *H*, and *W* denote the number of channels, height, and width, respectively. Input features are processed through deep and shallow branches with different receptive field scales. To minimize computational load, each branch employs a stack of 1×1 and 3×3 convolutions along multiple depth directions, with the deep-to-shallow branch ratio set at 3:1. The branches operate independently, except for the shared filtration module.

**(1) Filtration Module.** This module facilitates information exchange between the deep and shallow branches, $X_{1}\in R^{C\times H\times W}$ and $X_{2}\in R^{C\times H\times W}$, offering four operational modes: **None:** No modification is applied, and the output remains ($X_{1}$, $X_{2}$). **Exchange:** The features of the two branches are swapped, resulting in ($X_{2}$, $X_{1}$). **Gate:** The input data are first entered into the first fully connected layer, which transforms them linearly through weights and biases, capturing the primary characteristics of the data. Subsequently, using Relu layers enables the model to learn and simulate more complex data relationships, alleviating the problem of disappearing gradients. This is followed by a second fully connected layer, which further extracts and combines features to form a more complex and abstract representation of features. After activating the function, each input data point is finally multiplied by its corresponding transformed output element by element. This operation not only retains some characteristics of the original data but also reweights them by the learned weights, thus enhancing the response-ability of the model to the input characteristics. **Attention:** First, Xa and Xb are rearranged into query and key. By matrix multiplication, we calculate energya between Xa’s query and Xb’s key, and energyb between Xb’s query and Xa’s key. Energy represents the strength of the correlation between the different feature maps. Secondly, the output takes the maximum value and subtracts the original energy value to suppress the non-maximum value, and the softmax function is applied to obtain the normalized attention weight. The weighted feature representation is obtained by multiplying the attention weight by the projected value of the input feature map. Finally, the output is scaled by a learnable parameter and then added to the original input feature map to form a residual join. Residual connections help avoid the problem of disappearing gradients in deep networks, making deep networks easier to train.

**Figure 4:**
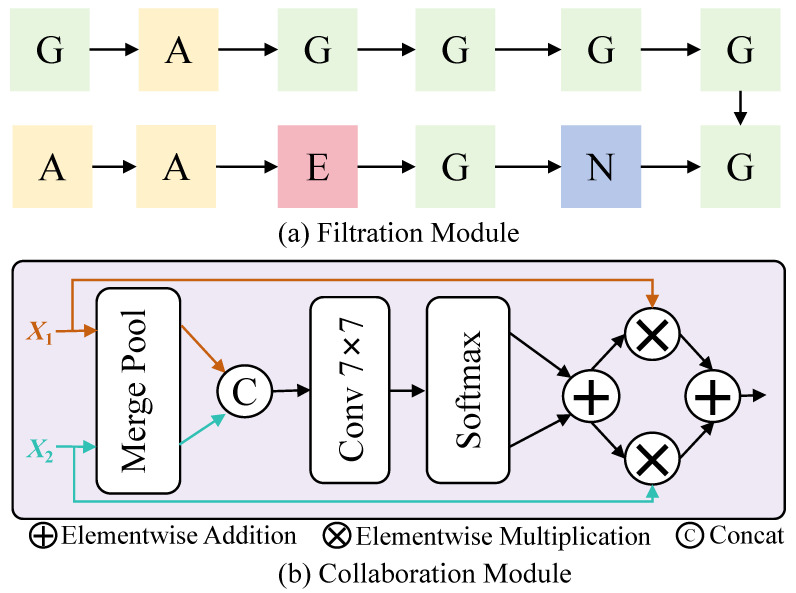
(**a**) The optimal detailed filtration module obtained in the CVNet architecture through NAS. N: none; E: exchange; G: gate; A: attention. (**b**) Collaboration module structure diagram. The 7×7 configuration is selected through cross-validation.

Ultimately, after the search process conducted by NAS, the optimal structure of CVNet, as depicted in Figure 4, is composed of the following: gate, attention, gate, gate, gate, gate, gate, none, gate, exchange, attention, and attention.

**(2) Collaboration Module.** After filtration, the deep and shallow branch features are fused through collaboration operations. Simple fusion methods, such as directly adding features, are prone to noise interference, making it difficult to achieve effective fusion. To address this, we propose a collaboration module for efficient feature fusion, illustrated in the purple area of Figure 4. Deep and shallow information is aggregated by merge pooling, which is composed of global average and max pooling. We concatenate the outputs of the two branches from the merge pooling to generate aggregated features. The aggregated features, meticulously selected through cross-validation, are passed through 7×7 convolution layers to evaluate input feature weights in the channel dimension. We then apply a Softmax function to normalize these weights to ensure they sum to 1, helping identify the most representative features. These weights are then applied to the features via multiplication, effectively fusing them to provide richer, more refined representations. This process enhances the network’s ability to capture common and joint features, leading to a more comprehensive scene understanding.

### 4.4. Loss Function

We use a common ReID loss combining softmax loss and triplet loss to enhance the network’s discriminative power. The softmax loss, promoting class separation, is defined as

(1) $$L_{\mathrm{softmax}}=-\sum_{i=1}^{N}y_{i}\log\left(\frac{e^{z_{i}}}{\sum_{j=1}^{C}e^{z_{j}}}\right),$$

where *N* is the number of samples, *C* the classes, $y_{i}$ the label, and $z_{i}$ the logit for the *i*-th sample. The triplet loss ensures that an anchor is closer to positives than negatives:

(2) $$L_{\mathrm{triplet}}=\max\left(0,\mathrm{margin}+d\left(a,p\right)-d\left(a,n\right)\right),$$

where d(a,p) and d(a,n) are distances, and margin defines minimum separation. The total loss combines both

(3) $$L_{\mathrm{total}}=L_{\mathrm{softmax}}+L_{\mathrm{triplet}}.$$

## 5. Experiment and Analysis

### 5.1. Implementation Details

The deep learning framework used in this work is PyTorch with FP16 training for enhanced computational efficiency. The model is trained for 350 epochs using a dual-optimizer configuration, where stochastic gradient descent (SGD) with a learning rate of 0.065 and momentum of 0.9 is employed for most parameters, while Adam with an initial learning rate of 0.002 is applied to specific components. A 10-epoch warm-up period is introduced to stabilize training by gradually increasing the learning rate. Images are uniformly resized to 256×256 for consistency, and a weight decay of $5\times 10^{-4}$ is implemented for regularization to prevent overfitting. A batch size of 64 is used to balance computational efficiency and effective model updates, with MSINet [28] serving as the baseline for all experiments.

### 5.2. Comparison with State-of-the-Art Methods

We perform extensive evaluations on the proposed CVPair v1.0 dataset, re-implementing both traditional and lightweight ReID methods, as shown in Table 2. Furthermore, we compare our approach on existing ReID datasets (VeRi-776 and VehicleID datasets), as detailed in Table 3.

**CVPair v1.0 Dataset Results.** We evaluate the performance of leading ReID models on the newly introduced CVPair v1.0 dataset, as shown in Table 2. For the A2G modality, our experiments report a mAP of 45.6% and a Rank1 accuracy of 67.2%, while the G2A modality achieves a mAP of 35.8% and a Rank1 accuracy of 53.9%. Compared to traditional ReID methods, CVNet, with only 4.4M parameters, outperforms the next-best model by 13.7% in mAP and 23.9% in Rank1 accuracy, demonstrating its remarkable efficiency in low-complexity scenarios. Furthermore, compared to lightweight methods, our approach achieves the best performance on CVPair v1.0. While it has slightly more parameters than StarNet-S1 + CH, the significant accuracy improvement underscores its competitiveness.

**Existing Datasets Results.** We evaluated state-of-the-art vehicle ReID methods on the VeRi-776 and VehicleID datasets, as shown in Table 3. Our method achieves Rank1 accuracies of 93.6% on VeRi-776 and 85.9% on VehicleID with only 4.4M parameters, outperforming all competitors. In comparison, Trans-ReID [32] (86.6M parameters) achieves 85.2%, while Vit-reid [38], GiT [39], and SOFCCT [37], with 57.3M parameters, achieve 80.5%, 84.7% and 77.8% on VehicleID, respectively. CAL [36] (23.8M parameters) achieves only 75.1%. These results highlight the superior efficiency and performance of our approach.

### 5.3. Ablation Studies and Analysis

**Role of the MSL and DFC.** We evaluate the contributions of the DFC and MSL modules on the CVPair v1.0 dataset by replacing them with ResNet50’s 7 × 7 convolutions (Res. 1) and Stage (Res. S), respectively. As shown in Table 4, incorporating DFC improves Rank1 accuracy by 1.9% and mAP by 1.2%, demonstrating its ability to bridge cross-view feature discrepancies by capturing both shallow and deep features. Its filtration mechanism enhances feature interaction, preserving feature richness and boosting robustness to perspective variations. Similarly, the MSL module improves Rank1 accuracy by 3.8% and mAP by 5.1% by integrating Inception layers, depthwise convolutions, and lightweight attention blocks to efficiently capture multi-scale features. With only 0.09 M additional parameters, MSL delivers significant performance gains with minimal computational overhead, making it ideal for resource-constrained scenarios.

**Figure 5:**
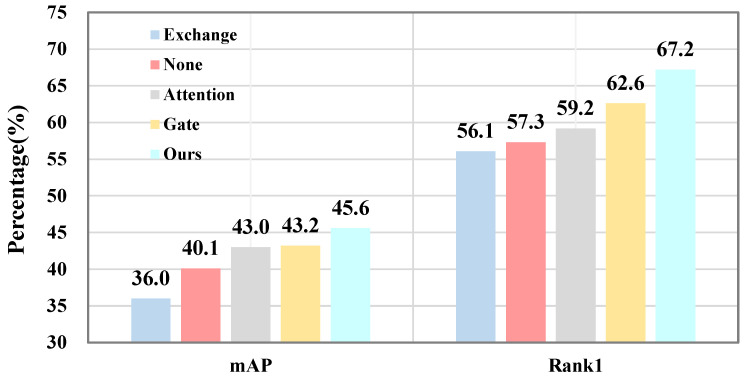
Comparison of the performance of different NAS strategies.

**Impact of NAS Strategies.** Figure 5 compares filtration strategies in the DFC module, with “Ours” representing NAS-optimized operations. Strategies like “None” and “Exchange” perform poorly due to the lack of trainable parameters. Gating and attention operations improve performance by enhancing feature exchange, but CVNet achieves the best results by organizing interactions effectively. The NAS-optimized DFC achieves a 9.6% mAP gain and a 11.1% Rank1 improvement over “None”, demonstrating its effectiveness.

**Impact of *n*.** Table 5 shows that a stage (n=3) configuration achieves the highest Rank1 and mAP scores. Fewer stages limit feature extraction, reducing the model’s ability to capture complex patterns, while more stages risk overfitting due to excessive parameters. The n=3 configuration strikes a balance between depth and computational efficiency, enabling effective learning of discriminative features. Therefore, this work adopts n=3 as the default setting.

**Figure 6:**
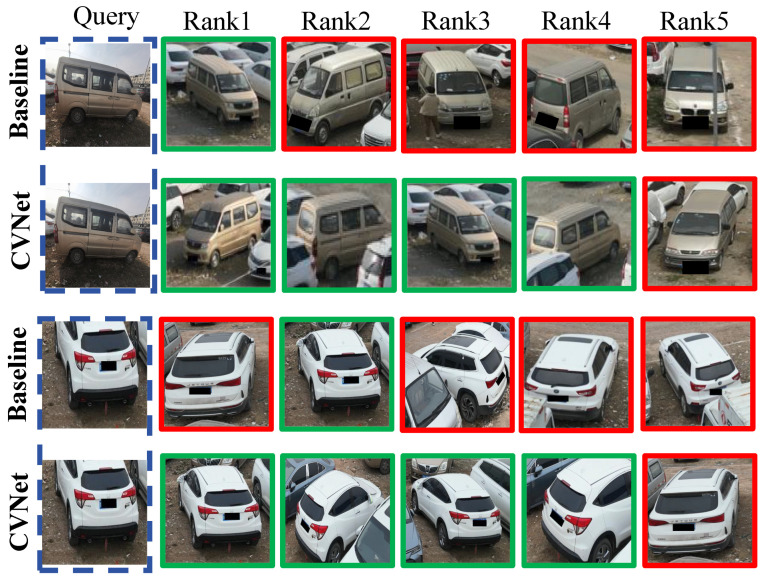
Visualization of CVNet and baseline. Green boxes indicate correct matches, while red boxes denote errors. **CVNet delivers more accurate retrieval results**.

**Visualization Results.** Figure 6 illustrates the top-5 retrieval results for both the baseline and CVNet models in the CVPair v1.0 dataset, highlighting CVNet’s superior accuracy and ability to capture fine-grained vehicle details. Compared to the baseline, CVNet demonstrates improved retrieval precision, correctly identifying subtle features that the baseline model often misses. Using MSL and DFC modules, along with multi-scale feature extraction and information fusion, CVNet enhances regional localization and scene understanding, leading to more accurate and robust vehicle differentiation across diverse perspectives.

## 6. Conclusions

We propose CVNet, a lightweight network designed for cross-view vehicle ReID, addressing challenges of scale variation and computational constraints. The MSL module extracts multi-scale features with localization attention, while the DFC module employs a dual-branch design to capture unique and shared cross-view features. Along with CVNet, we introduce CVPair v1.0, the first benchmark for cross-view ReID, featuring 14,969 images of 894 vehicle identities. CVNet achieves state-of-the-art performance on CVPair v1.0, VehicleID, and VeRi-776, advancing cross-view ReID research.

## Figures and Tables

**Table 1:** Comparison of our dataset CVPair v1.0 with the public dataset. Our datasets were compared with existing public ground and aerospace datasets. At present, Aerial–Ground only has ReID datasets for people.

Datasets	Ground–Ground		Aerial–Aerial				Ground–Aerial
	VeRi-Wild [18]	VehicleID [19]	VRAI [20]	VeRi-UAV [21]	VRU [22]	UAV-VeID [23]	CVPair v1.0
Images	416,314	221,567	137,613	17,515	172,137	41,917	14,969
Views	fixed	fixed	mobile	mobile	mobile	mobile	fixed & mobile
Platforms	CCTV	CCTV	UAV	UAV	UAV	UAV	UAV & Phone
Altitude	<10 m	<10 m	15–80 m	10–30 m	15–60 m	15–60 m	3–13 m
UAVs	0	0	2	1	5	2	1
Target	Vehicle	Vehicle	Vehicle	Vehicle	Vehicle	Vehicle	Vehicle
Task	Query and gallery from ground views.		Query and gallery from aerial views.				Query and gallery from ground and aerial views.

**Table 2:** Performance (%) comparison on CVPair v1.0. ‘CH’ indicates a classification head. Methods marked with ‘*’ are our re-implemented methods. The best performance is highlighted in bold.

	Models	A2G			G2A			#Params (M) ↓	FPS ↑
		mAP	Rank1	Rank5	mAP	Rank1	Rank5		
Traditional ReID Methods	* PPLR [29]	12.6	15.1	37.2	7.0	10.9	22.3	26.8	3.1
	* MGN [30]	26.8	23.9	66.0	29.7	29.6	49.7	70.4	1.2
	* BoT [31]	31.6	43.3	69.8	24.1	35.2	58.1	23.8	3.5
	* Trans-ReID [32]	31.9	42.1	73.0	28.5	38.8	59.4	86.6	1.0
Lightweight Methods	* StarNet-S1 + CH [33]	14.8	17.1	39.4	11.8	14.5	25.3	**3.2**	17.8
	* MobileOne-S1 + CH [34]	17.2	21.0	48.2	20.4	22.9	38.1	5.1	16.2
	* SBCFormer-XS + CH [35]	19.4	24.9	52.6	23.8	25.1	42.2	5.9	15.4
	* FasterNet-T1 + CH [8]	28.8	39.2	65.1	27.7	28.5	46.3	7.9	13.7
	**CVNet (Ours)**	**45.6**	**67.2**	**88.1**	**35.8**	**53.9**	**76.3**	4.4	**18.2**

**Table 3:** Performance on VeRi-776 and VehicleID datasets.

Datasets	Method	#Params (M) ↓	Rank1 ↑
VehicleID [19]	CAL [36]	23.8	75.1
	SOFCT [37]	57.3	77.8
	Vit-reid [38]	57.3	80.5
	GiT [39]	57.3	84.7
	Trans-ReID [32]	86.6	85.2
	**Ours**	**4.4**	**85.9**
VeRi-776 [40]	PAMTRI [41]	10.0	71.9
	Trans-ReID [32]	86.6	85.2
	CAL [36]	23.8	85.9
	KPGST [42]	11.7	92.4
	**Ours**	**4.4**	**93.6**

**Table 4:** Ablation studies on the proposed CVPair v1.0 dataset.

Methods	mAP	Rank1	Rank5
Baseline	39.4	54.3	82.5
Res. 1 → MSL	44.5	58.1	83.5
Res. S → DFC	40.6	56.2	82.7
**Ours**	**45.6**	**67.2**	**88.1**

**Table 5:** Effect of the number of stages *n* on CVNet performance.

*n*	mAP	Rank1	Rank5
1	33.2	46.0	76.1
2	36.5	50.3	77.9
**3**	**45.6**	**67.2**	**88.1**
4	32.6	44.7	72.2

## Data Availability

Data are contained within the article.
